# The Good Life program for healthy brain aging is a new standard of care in California

**DOI:** 10.1002/alz.093539

**Published:** 2025-01-09

**Authors:** David K Johnson

**Affiliations:** ^1^ UC Davis, Walnut Creek, CA USA

## Abstract

**Background:**

In 2022, *The Good Life* program for healthy brain aging (**TGL**) was recognized by Governor Newsom and his *Task Force on Alzheimer’s Prevention and Preparedness* as a new Standard of Care for Alzheimer’s disease preventive medicine in California. TGL is a large geriatric focused public health initiative that promotes healthy lifestyles to older adults from all walks of life. It is made up of a series of three lifestyle intervention (**LSI**) classes that are produced and delivered on a video conferencing platform. The *Let’s Get Moving* (**LGM**) series is a live, virtual exercise and fitness class delivered to seniors several times per week by a certified fitness trainer using the Exercise is Medicine curriculum of the American College of Sports Medicine. The *Let’s Get Cooking* (**LGC**) series delivers virtual healthy cooking classes to seniors in their own home by our staff trained in the 18 Reasons cooking curriculum, an empirically validated nutrition program that uses hands on kitchen training to promote caloric restriction and healthier meal replacement. The peer‐to‐peer mental health TGL program applies a group therapy‐like formatted virtual meeting series to promote mindfulness and emotional literacy.

**Method:**

TGL implemented a 2‐arm pragmatic clinical trial (without random assignment) to test if high versus low levels of Perceived Social Support, Perceived Positive Health Change, and Anxiety symptoms affect attendance in TGL’s intervention over an 18‐week repeated measures follow up. The study is in its 2^nd^ year and will run over 4 years with quarterly assessments of the 3 co‐primary outcomes. Each participant is observed up to 18 months, yielding up to 6 repeated measures of these outcomes per individual.

**Result:**

TGL is very successful at engaging BA and LA in healthy lifestyle classes. TGL has over 2,000 older adult members (approximately 70% of our membership are either BA or LA older adults).

**Conclusion:**

TGL’s success strongly indicates that virtual classes, which eliminates the burdens for older adults to attend in‐person classes, are effective in increasing adoption of healthy lifestyle behaviors.

